# Concurrent Alcohol and Tobacco Dependence: Mechanisms and Treatment

**Published:** 2002

**Authors:** David J. Drobes

**Affiliations:** David J. Drobes, Ph.D., is an associate professor at the Moffitt Cancer Center & Research Institute, University of South Florida, Tampa, Florida

**Keywords:** comorbidity, AODD (alcohol and other drug dependence), alcoholic beverage, tobacco in any form, nicotine, smoking, genetic linkage, cross-tolerance, AOD (alcohol and other drug) sensitivity, neurotransmitters, brain reward pathway, cue reactivity, social AODU (AOD use), cessation of AODU, treatment outcome, combined modality therapy, literature review

## Abstract

People who drink alcohol often also smoke and vice versa. Several mechanisms may contribute to concurrent alcohol and tobacco use. These mechanisms include genes that are involved in regulating certain brain chemical systems; neurobiological mechanisms, such as cross-tolerance and cross-sensitization to both drugs; conditioning mechanisms, in which cravings for alcohol or nicotine are elicited by certain environmental cues; and psychosocial factors (e.g., personality characteristics and coexisting psychiatric disorders). Treatment outcomes for patients addicted to both alcohol and nicotine are generally worse than for people addicted to only one drug, and many treatment providers do not promote smoking cessation during alcoholism treatment. Recent findings suggest, however, that concurrent treatment for both addictions may improve treatment outcomes.

Alcohol consumption and tobacco use are closely linked behaviors. Thus, not only are people who drink alcohol more likely to smoke (and vice versa) but also people who drink larger amounts of alcohol tend to smoke more cigarettes. Furthermore, patients diagnosed with dependence on one of the drugs also are commonly diagnosed with dependence on the other drug (e.g., Zacny 1990). In fact, smoking rates among alcoholics have been estimated to be as high as 90 percent, with approximately 70 percent of alcoholics smoking at least one pack of cigarettes per day (National Institute on Alcohol Abuse and Alcoholism 1998). Similarly, smokers are far more likely to consume alcohol than are nonsmokers, and smokers who are dependent on nicotine have a 2.7 times greater risk of becoming alcohol dependent than nonsmokers (e.g., Breslau 1995). Finally, although the smoking rate in the general population has gradually declined over the past three decades, the smoking rate among alcoholics has remained persistently high (e.g., Hays et al. 1999).

Concerns about the concurrent use of alcohol and tobacco are particularly salient given the detrimental impact of this drug combination on the individual and on society. For instance, alcohol and tobacco when used together increase the risk of various forms of cancer (e.g., mouth and esophageal cancer), as well as cardiovascular disease, more than use of either drug alone (e.g., U.S. Department of Health and Human Services 1989). The concurrent use of both drugs by pregnant women can also result in more severe prenatal damage and neurocognitive deficits in their offspring than use of either drug alone (e.g., Martin et al. 1997). Furthermore, the combined use of alcohol and tobacco among adolescents is more predictive of illicit drug use and various personal and social problems among this population than use of either drug alone (e.g., Hoffman et al. 2001).

Given the frequent occurrence and broad implications of concurrent alcohol and tobacco use, research and clinical efforts clearly must focus on people who abuse both drugs. Over the past decade, interactions between alcohol and tobacco have indeed received growing attention from both basic and clinical researchers. Alcohol dependence and smoking, individually and in combination, are complex forms of addictive behavior that may be influenced by a variety of genetic, neurobiological, conditioning, and psychosocial mechanisms, as described in this article. In addition to these mechanisms, the article discusses issues related to the treatment of alcoholic smokers. This overview will necessarily be selective; for instance, there is little mention of sociocultural (e.g., economic and demographic) factors that also may contribute to concurrent use of alcohol and tobacco (see Bobo and Husten 2000).

## Mechanisms Underlying Combined Alcohol and Tobacco Use

### Genetic Factors

The importance of genetic influences on both alcoholism and smoking has gained widespread recognition over the past decade. Using behavioral genetic methods, such as twin and adoption studies, as well as genetic epidemiological approaches, researchers have established that both alcoholism and smoking have strong heritable components (e.g., Prescott and Kendler 1995). In general, heritability, which estimates the proportion of variability within an observed characteristic that can be attributed to genetic factors, appears to be slightly higher for smoking-related variables (e.g., smoking initiation and smoking persistence) than for alcoholism (e.g., Heath and Madden 1995). Moreover, several researchers have indicated that a substantial shared genetic risk exists between smoking and alcoholism— that is, genetic factors that increase the risk for smoking also increase the risk for alcoholism and vice versa (e.g., Koopmans et al. 1997; Prescott and Kendler 1995).

The relative contributions of genetic and environmental risk factors may depend on a person’s age and gender. Thus, one study found that the combined risk for alcohol use and smoking in adolescents was primarily attributable to shared environmental features (e.g., peer influences) whereas in young adults, this risk was significantly influenced by genetic factors (Koopmans et al. 1997). Laboratory findings suggest that reduced subjective effects of alcohol (e.g., euphoria or sedation) among smokers may underlie this genetic association (Madden et al. 1997), particularly among women.

Recent molecular genetic studies have attempted to identify specific genetic factors that may underlie various forms of addictive behavior. Perhaps the strongest evidence for individual genes that may contribute to both smoking and alcoholism involves the dopaminergic reward system. Dopamine is a brain chemical (i.e., neurotransmitter) that mediates the communication among brain cells in certain brain regions. Some of these brain regions play a role in the pleasant (i.e., rewarding) effects of drugs such as alcohol and nicotine. To exert its effects, dopamine released by one brain cell interacts with specific protein molecules (i.e., receptors) on the surface of neighboring cells, and this interaction causes a biochemical reaction in those cells. Some evidence suggests that certain variants of genes that regulate the activity of dopamine or its receptors may be related to the risk of excessive alcohol consumption or smoking (e.g., Lerman et al. 1999; Li 2000). The results at this stage are merely suggestive, but the application of molecular genetic research techniques to studies of complex behaviors such as alcohol and nicotine addiction is progressing rapidly and may yield important findings within the next decade.

One development that most likely will accelerate researchers’ understanding of genetic factors contributing to alcoholism and smoking will be the establishment of valid and reliable endophenotypes for these addictive behaviors. An endophenotype is an objective and measurable characteristic of a person that is thought to be more directly related to the person’s genetic makeup (i.e., genotype) than are typical diagnostic categorizations (e.g., alcohol abuse or dependence). Perhaps the best-established example of such an endophenotype in the drug addiction field is the P300 component of the event-related brain potential (ERP). ERPs are brain waves elicited by a sudden stimulus (e.g., a light or sound). One component of an ERP typically can be measured approximately 300 milliseconds after the stimulus occurs and is therefore called the P300 signal. It is thought to represent cognitive, or attentional, processing of novel information. This P300 signal commonly is reduced in size in people at risk for alcoholism (e.g., Porjesz et al. 1998). Recent work has also shown that smokers may exhibit ERPs with a reduced P300 signal (e.g., Anokhin et al. 2000). By replicating these findings and identifying additional valid endophenotypes for alcoholism and smoking, researchers hope to detect stronger relationships between these forms of addictive behavior and certain genes. In addition, these studies may lead to a fuller understanding of the mechanisms through which these genes influence behavior.

### Neurobiological Mechanisms

Several neurobiological mechanisms may underlie the strong relationship between alcohol and tobacco use. Both the ability of one drug to reduce the effects of the other drug (i.e., cross-tolerance) and the ability of one drug to increase the effects of the other drug (i.e., cross-reinforcement) may play important roles in mediating this relationship (Pomerleau 1995). Such processes could act immediately when alcohol and nicotine are taken together, or they could involve changes in nerve cell function that occur over time with repeated usage of either one or both drugs. It is also possible that the two drugs when taken together create a combined reward effect that is qualitatively different from the effects of either drug taken alone.

The development of tolerance (and, by extension, cross-tolerance) to both pleasurable and aversive drug effects is thought to support the development or maintenance of an addiction. Thus, tolerance to pleasurable drug effects requires the user to consume increasing drug amounts to achieve the desired rewarding effects. Conversely, tolerance to aversive drug effects enables the user to experience pleasant effects while not experiencing the initial aversive drug effects. Experimental evidence of cross-tolerance between alcohol and nicotine comes from several lines of research. For instance, mice bred for different levels of sensitivity to certain alcohol effects (e.g., either extremely high or extremely low sensitivity to alcohol’s sedative effects) exhibit corresponding changes in their behavioral and physiological responses to nicotine (Collins 1990). Also, mice that chronically receive nicotine (via intravenous infusion) or alcohol (via a liquid diet) show cross-tolerance to a drug-induced decrease in body temperature when the alternate drug is given (Collins et al. 1988). Finally, in a recent study, female adolescent mice treated with alcohol for 4 days displayed cross-tolerance to nicotine’s effects on body temperature and activity when they were tested 30 days later (Lopez et al. 2001).

Extending these demonstrations of cross-tolerance from animal models to the phenomenon of concurrent alcohol and nicotine dependence in humans, one could hypothesize that people who regularly consume both alcohol and nicotine may develop dependence on both drugs more rapidly than if they consumed only one drug, because the rate of tolerance development would be increased. Alternatively, smoking may promote alcohol consumption through an immediate (i.e., acute) form of cross-tolerance. This means that smokers may be able to consume more alcohol because nicotine exerts a stimulatory effect that can directly counteract both the sedative properties of alcohol and the cognitive deficits associated with alcohol intoxication. This hypothesis is supported by findings that nicotine administration directly increases alcohol consumption in animal models; this effect appears to be mediated through receptors for nicotine in the brain (e.g., Lê et al. 2000). Similarly, earlier laboratory studies with humans showed that alcohol consumption increased the amount and rate at which participants smoked cigarettes (e.g., Mello et al. 1980).

**Figure:**
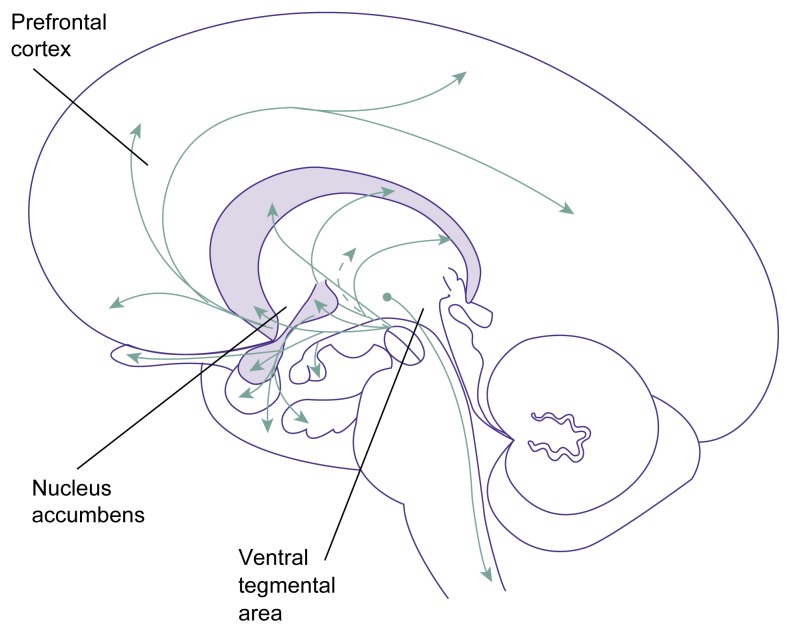
Dopaminergic pathways in the brain, including the mesolimbic dopaminergic system, which consists of the ventral tegmental area, the nucleus accumbens, and the prefrontal cortex. This system has been implicated in the motivation to obtain various rewards, including the positive reinforcement of alcohol and nicotine. SOURCE: Adapted from Heimer, L. *The Human Brain and Spinal Cord: Functional Neuroanatomy and Dissection Guide*. 2d ed. New York: Springer-Verlag, 1995.

As mentioned earlier, components of the brain signaling system involving the neurotransmitter dopamine may play a role in the genetic basis for both alcohol and tobacco addiction. One brain system that uses dopamine as a primary neurotransmitter is the mesolimbic dopamine system, which has been implicated in the motivation to obtain various rewards, including alcohol and nicotine (e.g., Wise 1988). This system encompasses several brain regions, most notably the ventral tegmental area, the nucleus accumbens, and the prefrontal cortex (see figure). Both alcohol and nicotine directly activate dopamine-releasing nerve cells within the ventral tegmental area, ultimately leading to dopamine release in the nucleus accumbens and prefrontal cortex. These pathways appear to become sensitized with repeated use of either drug, a process called neuroadaptation (e.g., Robinson and Berridge 1993). One theoretical model called the incentive sensitization model (Robinson and Berridge 1993) posits that stimuli which have been closely associated with prior drug use (e.g., the sight of a beer bottle or a pack of cigarettes) gradually become more powerful (i.e., gain incentive salience) because of this sensitization. According to this model, both alcohol- and nicotine-associated stimuli may activate the mesolimbic dopaminergic system in people who frequently use both drugs, thereby increasing the overall vulnerability to an addiction in those people.

Another brain neurotransmitter system that may be involved in alcohol-tobacco interactions is the endogenous opiate system. Endogenous opiates are molecules produced naturally by the body that have effects similar to opiates (e.g., morphine). Alcohol appears to stimulate the endogenous opiate system, which may contribute to alcohol’s pleasurable effects. Clinical and laboratory-based studies have shown that agents that block the effects of endogenous opiates (i.e., opiate antagonists) can reduce alcohol consumption (see Anton 2001). The effects of nicotine may also be partly mediated through this opiate brain system, although studies on the effects of opiate antagonists on smoking behavior and other nicotine-related responses have provided equivocal results to date.

### Conditioning Mechanisms

It is a common observation that people who drink alcohol and smoke tend to engage in these behaviors in particular situations (e.g., in a bar or at a party) and contemporaneously. Furthermore, studies have confirmed that relapse to smoking following smoking cessation is strongly associated with alcohol consumption (e.g., Brandon et al. 1990). These observations support the hypothesis that alcohol and smoking may become associated through a process called cue conditioning because of the frequent concurrent use of the two drugs. In general, conditioning models of addiction suggest that cues previously paired with drug use (e.g., the sight of a liquor bottle or the smell of a lighted cigarette) will elicit conditioned responses, including cravings and associated physiological activity. These cue-elicited cravings and physiological reactions, in turn, can motivate ongoing drug use and increase the probability of relapse among people who are abstinent (e.g., Drobes and Tiffany 1997). Numerous laboratory studies have supported this view, demonstrating that various alcohol and smoking-related cues can elicit cravings and physiological responses among alcoholics and smokers, respectively (for a review, see Carter and Tiffany 1999).

Several human laboratory studies suggest a role for cue conditioning in the close association between alcohol use and smoking. For instance, one study showed that the severity of nicotine dependence among alcoholic smokers was related to the strength of alcohol cravings elicited by alcohol cues (Abrams et al. 1992). Other findings have demonstrated that alcohol cues can simultaneously increase smoking urges and alcohol urges among alcoholic smokers (e.g., Gulliver et al. 1995; Rohsenow et al. 1997). Overall, laboratory findings suggest that substantial overlap between alcohol and smoking cues may exist in promoting drug cravings and drug consumption—that is, both types of cues may elicit cravings and consumption of either drug.

Even the administration of alcohol or nicotine can serve as a conditioned pharmacological or sensory cue. Accordingly, research that evaluates the effects of the administration of either drug on responding to the other drug can help determine the role of conditioning factors in concurrent alcohol and tobacco use. For instance, several early studies demonstrated that alcohol consumption can promote smoking (e.g., Mello et al. 1980). Furthermore, a more recent study of nonalcoholic smokers showed that cue-elicited cravings for nicotine increased when the participants first consumed alcohol (Burton and Tiffany 1997). However, craving increases in this study were not specific to smoking-related cues, which implies that alcohol consumption leads to a more general increase in cravings to smoke. Finally, another laboratory study investigated how hard people who had been allowed to smoke or who were smoking deprived would work on a computer task to receive alcohol.^1^ Each participant was tested in two separate sessions involving either ad lib smoking or smoking deprivation prior to the session. During each session, the task was performed twice, both before and after receiving a standard dose of alcohol. The study found that after the men had received a standard dose of alcohol, those who had been allowed to smoke before working on the task worked harder to obtain more alcohol than did men who had been deprived of nicotine overnight (Perkins et al. 2000). This effect was not observed when the men were tested before they had received the alcohol, indicating that when it is combined with alcohol consumption, nicotine consumption can increase the motivation to drink alcohol. This interaction between nicotine and alcohol consumption was not observed in women, suggesting that important gender differences may exist with respect to pharmacological and motivational influences on alcohol and tobacco use.

Research has not yet directly examined acute nicotine effects on reactivity to alcohol cues, nor has the combined impact of alcohol and nicotine administration on cue-elicited craving, drug effects, or drug consumption been studied extensively (for a review, see Perkins 1997). Overall, the available research suggests that alcohol and nicotine can have interactive effects on the motivation to consume either drug. Further research is needed to obtain a better understanding of the interactive effects of various pharmacological and cue manipulations on cravings for and consumption of alcohol and nicotine.

### Psychosocial Factors

Even at the earliest stages of drug use, which often occurs during adolescence, common psychosocial factors may promote the use of both alcohol and tobacco. For instance, personality characteristics that remain stable throughout a person’s life often have been implicated as playing a role in the initiation of both alcohol and tobacco use (e.g., Flay et al. 1995). These characteristics may include sensation seeking, impulsivity, compulsiveness, and neuroticism (i.e., trait anxiety). Such a role of personality characteristics in determining alcohol consumption and smoking is not incompatible with the genetic mechanisms discussed above. Indeed, many of these personality variables are themselves heritable, and the genetic risk for alcohol use and smoking may be mediated partly through these personality traits.

Another important psychosocial influence on the initiation of combined alcohol and tobacco use stems from family modeling. Thus, numerous studies show that adolescents who are exposed to older family members who smoke and drink are more liable to engage in these behaviors than are adolescents without such family members (e.g., Bobo and Husten 2000). Accordingly, combined alcohol and tobacco use may become a self-propagating cycle across familial generations independent of any direct genetic influence.

Other important modeling influences for alcohol use and smoking behaviors are likely to be peer related. As mentioned earlier, the impact of genetic factors on alcohol use and smoking may be somewhat less pronounced during adolescence than during early adulthood (Koopmans et al. 1997). This observation is consistent with findings supporting a strong role of parental and peer influences (e.g., Hoffman et al. 2001). Furthermore, both alcohol use and smoking commonly serve as outlets for adolescent rebelliousness, and both behaviors are associated with illicit drug use and other problems among adolescents (Hoffman et al. 2001).

Temporary psychological states in otherwise mentally healthy people also may contribute to the ongoing use of alcohol and tobacco. For example, both laboratory and field studies have found that situational stress and negative emotional states (e.g., anxiety and depression) can serve as cues that elicit alcohol or tobacco craving or consumption of these drugs in active drinkers or smokers (e.g., Tiffany and Drobes 1990). People also may use alcohol and nicotine to alleviate stress or tension. Indeed, both drugs exhibit extreme versatility in their ability to regulate mood, in that they may be used either to help a person relax or to stimulate or energize the person. Alcohol and smoking also both frequently serve as “social lubricants” in social situations.

Rates of alcohol and tobacco consumption are disproportionately high among people with comorbid psychological disorders, particularly various affective (e.g., depression) and anxiety disorders. People with such disorders presumably use alcohol and tobacco to self-medicate their affective symptoms through the direct stimulatory or stress-reducing drug effects. The order in which the psychological disorders and alcohol and tobacco use develop, however, is not always clear and may vary for different people. Thus, alcohol and tobacco use may represent a form of (maladaptive) coping with a preexisting psychological disorder or they may precede or exacerbate the development of psychopathology. Finally, alcohol and tobacco use may be part of a broader constellation of symptoms associated with the comorbid condition. Further long-term studies with alcohol and tobacco users who exhibit or later develop various forms of psychopathology may clarify the causal pathways underlying these relationships.

Several other psychosocial variables have been tied theoretically or empirically to the risk of combined alcohol and tobacco use in various situations. These variables include life stressors (e.g., loss of a job or a loved one), social support, self-efficacy, coping skills, and expectancies (i.e., expectations about the effects of alcohol and tobacco on behavior). These and other psychosocial factors most likely interact with genetic, biological, and conditioning mechanisms in unique ways throughout each person’s history of alcohol and nicotine use, including initiation, maintenance, cessation, and relapse, to determine that person’s risk of alcohol and nicotine abuse and dependence.

## Treatment of Smoking in Alcoholics

For people addicted to alcohol and nicotine, outcomes during treatment for alcoholism, smoking, or both are often less favorable than for people addicted to only one drug. For example, alcoholics who smoke generally are less successful in achieving and maintaining sobriety than are nonsmoking alcoholics (e.g., Hughes 1995). Furthermore, in alcoholics treated for both addictions, relapse to smoking is considered a risk factor for alcohol relapse (e.g., Johnson and Jennison 1992). Similarly, nicotine dependence and the experience of nicotine withdrawal appear to be more severe in smokers with a history of alcohol dependence (e.g., Marks et al. 1997), and rates of successful smoking cessation are lower among smokers with past or current alcohol problems (e.g., DiFranza and Guerrera 1990). Consequently, improvements in treatment outcomes among smoking alcoholics remain an important challenge for the future.

Until recently, treatment providers generally believed that smoking cessation was contraindicated during alcoholism treatment for several reasons. Some alcoholism program philosophies considered smoking a relatively benign problem compared with alcohol dependence. Another reason was the fear that smoking cessation would lead to poorer alcoholism treatment outcomes, either by increasing the clients’ stress or decreasing the effort that clients could devote to achieving abstinence from alcohol (e.g., Bobo and Gilchrist 1983). Finally, many alcoholism treatment providers believed that smoking serves as an effective coping tool for dealing with alcohol cravings and with the stress associated with alcohol withdrawal or protracted abstinence. Consequently, these providers were unwilling to take away that coping tool.

Despite longstanding fears from treatment providers that smoking cessation would interfere with alcoholism treatment, there are several reasons to anticipate that combined treatment for both addictions may lead to more favorable outcomes for both drugs. First, at a neurobiological level, alcohol and nicotine act, at least in part, on the same brain pathways involved in reward and craving. Therefore, it may be advantageous to cease using both drugs to reverse the effects on these pathways. One important caveat here is that nicotine appears to serve an acute protective function concerning certain neurotoxic effects of alcohol withdrawal. Therefore, extreme caution must be exercised in determining the optimal sequence of drug removal for patients desiring treatment for both addictions. Second, as discussed above, continued smoking or alcohol use may elicit or exacerbate craving for the other drug. Third, behavioral treatments based on coping-skill attainment may be more effective when developing skills are generalized to both types of addictive behavior. For instance, people may be able to obtain more practice at using coping skills if they apply them to both alcohol consumption and smoking. And fourth, a treatment approach that encourages an overall milieu of healthy lifestyle changes would be more generally consistent with abstinence from both drugs.

Another reason to support concurrent treatment for smoking and alcoholism is that more alcoholics will die from smoking-related illnesses than from alcohol-related causes (e.g., Hurt et al. 1996). The numerous problems associated with smoking are well documented, and most alcoholics entering treatment are aware of these problems and appear quite willing to receive concurrent smoking cessation treatment (e.g., Saxon et al. 1997). Even without formal smoking cessation treatment, smoking rates appear to decrease and the motivation to quit smoking increases following successful alcoholism treatment (e.g., Monti et al. 1995). Researchers have begun to evaluate the effectiveness of explicit smoking cessation attempts during alcoholism treatment as well as the impact of such attempts on the outcome of the alcoholism treatment (e.g., Martin et al. 1997). Findings to date generally do not confirm the traditional notion that only one addiction should be treated at a given time. It is still too early to tell what treatment configuration will be most effective for smoking alcoholics.

In concert with recent advances in their understanding of neurobiological factors that contribute to the development and maintenance of addictive behavior, including alcoholism and smoking, researchers have been exploring potentially useful pharmacological treatments that may benefit various types of addiction. Because it is likely that alcohol and nicotine act at least partially through the same brain reward pathways, it is reasonable to expect some overlap in the types of pharmacological treatments that may be effective in the treatment of alcoholism and smoking. For instance, as mentioned earlier, endogenous opioids play a role in mediating alcohol’s effects, and opiate antagonist medications (e.g., naltrexone and nalmefene) can be effective for the treatment of alcoholism. Recent studies have suggested that opiate pathways may also be involved in nicotine dependence (e.g., Pomerleau 1998). However, the usefulness of opiate antagonists as a treatment for smokers in general or alcoholic smokers in particular remains to be determined.

## Conclusions

Alcohol and tobacco use are highly correlated behaviors. For example, people who drink are very likely to smoke and vice versa; furthermore, people who are dependent on alcohol also are frequently dependent on nicotine. The costs of the combined use of these drugs to both the individual and society are substantial. Several potential mechanisms may promote the combined use of alcohol and nicotine. Although researchers have made substantial progress in delineating factors that may underlie alcohol and tobacco comorbidity, several research gaps remain. For example, investigators and clinicians still need to fully elucidate and consider the roles of various genetic, neurobiological, conditioning, and psychosocial factors in developing a more thorough understanding of this dual addiction. Important potential gender differences in how these mechanisms operate also merit further research, as do potential differences in the treatment of male and female alcoholic smokers.

Despite long-held views that smoking cessation attempts should be deferred or discouraged among alcoholics undergoing treatment, researchers have begun to evaluate treatment programs designed to address alcohol and nicotine dependence simultaneously. Early results of these analyses are promising, although additional research is clearly needed to optimize treatment outcomes and to address important health and safety concerns. The development of treatment programs for people dependent on both alcohol and nicotine will be greatly enhanced if such programs are based on a fundamental understanding of mechanisms that promote this dual addiction. Similarly, basic researchers should consider the clinical phenomenology of concurrent alcohol and tobacco use as a guiding force for investigating dual addictions in the laboratory.

## Footnotes

1 The effort a person puts into the task (i.e., how hard he or she works) in order to receive alcohol is considered an indication of his or her motivation to drink.

## References

[b1-136-142] Abrams DB, Rohsenow DJ, Niaura RS (1992). Smoking and treatment outcome for alcoholics: Effects on coping skills, urge to drink, and drinking rates. Behavior Therapy.

[b2-136-142] Anokhin AP, Vedeniapin AB, Sirevaag EJ (2000). The P300 brain potential is reduced in smokers. Psychopharmacology.

[b3-136-142] Anton RF (2001). Pharmacologic approaches to the management of alcoholism. Journal of Clinical Psychiatry.

[b4-136-142] Bobo JK, Gilchrist LD (1983). Urging the alcoholic client to quit smoking cigarettes. Addictive Behaviors.

[b5-136-142] Bobo JK, Husten C (2000). Sociocultural influences on smoking and drinking. Alcohol Research & Health.

[b6-136-142] Brandon TH, Tiffany ST, Obremski KM, Baker TB (1990). Postcessation cigarette use: The process of relapse. Addictive Behaviors.

[b7-136-142] Breslau N (1995). Psychiatric comorbidity of smoking and nicotine dependence. Behavior Genetics.

[b8-136-142] Burton SM, Tiffany ST (1997). The effect of alcohol consumption on craving to smoke. Addiction.

[b9-136-142] Carter BL, Tiffany ST (1999). Meta-analysis of cue-reactivity in addiction research. Addiction.

[b10-136-142] Collins AC, Galanter M (1990). Interactions of ethanol and nicotine at the receptor level. Recent Developments in Alcoholism: Vol. 8. Combined Alcohol and Other Drug Dependence.

[b11-136-142] Collins AC, Burch JB, de Fiebre CM, Marks MJ (1988). Tolerance to and cross tolerance between ethanol and nicotine. Pharmacology, Biochemistry, and Behavior.

[b12-136-142] DiFranza JR, Guerrera MP (1990). Alcoholism and smoking. Journal of Studies on Alcohol.

[b13-136-142] Drobes DJ, Tiffany ST (1997). Induction of smoking urge through imaginal and in vivo procedures: Physiological and self-report manifestations. Journal of Abnormal Psychology.

[b14-136-142] Flay BR, Petraitis J, Hu FB, Fertig JB, Allen JP (1995). Theory of triadic influence: Preliminary evidence related to alcohol and tobacco use. Alcohol and Tobacco: From Basic Science to Clinical Practice.

[b15-136-142] Gulliver SB, Rohsenow DJ, Colby SM (1995). Interrelationship of smoking and alcohol dependence, use and urges to use. Journal of Studies on Alcohol.

[b16-136-142] Hays JT, Schroeder DR, Offord KP (1999). Response to nicotine dependence treatment in smokers with current and past alcohol problems. Annals of Behavioral Medicine.

[b17-136-142] Heath AC, Madden PAF, Turner JR, Cardon LR, Hewitt JK (1995). Genetic influences on smoking behavior. Behavior Genetic Approaches in Behavioral Medicine.

[b18-136-142] Hoffman JH, Welte JW, Barnes GM (2001). Co-occurrence of alcohol and cigarette use among adolescents. Addictive Behaviors.

[b19-136-142] Hughes JR, Fertig JB, Allen JP (1995). Clinical implications of the association between smoking and alcoholism. Alcohol and Tobacco: From Basic Science to Clinical Practice.

[b20-136-142] Hurt RD, Offord KP, Croghan IT (1996). Mortality following inpatient addictions treatment. Role of tobacco use in a community-based cohort. JAMA: Journal of the American Medical Association.

[b21-136-142] Johnson KA, Jennison KM (1992). The drinking-smoking syndrome and social context. International Journal of the Addictions.

[b22-136-142] Koopmans JR, van Doornen LJ, Boomsma DI (1997). Association between alcohol use and smoking in adolescent and young adult twins: A bivariate genetic analysis. Alcoholism: Clinical and Experimental Research.

[b23-136-142] Lê AD, Corrigall WA, Watchus J (2000). Involvement of nicotine receptors in alcohol self-administration. Alcoholism: Clinical and Experimental Research.

[b24-136-142] Lerman C, Caporaso NE, Audrain J (1999). Evidence suggesting the role of specific genetic factors in cigarette smoking. Health Psychology.

[b25-136-142] Li T-K (2000). Pharmacogenetics of responses to alcohol and genes that influence alcohol drinking. Journal of Studies on Alcohol.

[b26-136-142] Lopez MF, White NM, Randall CL (2001). Alcohol tolerance and nicotine cross-tolerance in adolescent mice. Addiction Biology.

[b27-136-142] Madden PAF, Heath AC, Martin NG (1997). Smoking and intoxication after alcohol challenge in women and men: Genetic influences. Alcoholism: Clinical and Experimental Research.

[b28-136-142] Marks JL, Hill EM, Pomerleau CS (1997). Nicotine dependence and withdrawal in alcoholic and nonalcoholic ever-smokers. Journal of Substance Abuse Treatment.

[b29-136-142] Martin JE, Calfas KJ, Patten CA (1997). Prospective evaluation of three smoking interventions in 205 recovering alcoholics: One-year results of Project SCRAP-Tobacco. Journal of Consulting and Clinical Psychology.

[b30-136-142] Mello NK, Mendelson JH, Sellers ML, Kuehnle JC (1980). Effect of alcohol and marihuana on tobacco smoking. Clinical Pharmacology and Therapeutics.

[b31-136-142] Monti PM, Rohsenow DJ, Colby SM, Abrams DB, Fertig JB, Allen JP (1995). Smoking among alcoholics during and after treatment: Implications for models, treatment strategies, and policy. Alcohol and Tobacco: From Basic Science to Clinical Practice.

[b32-136-142] National Institute on Alcohol Abuse and Alcoholism (NIAAA) (1998). Alcohol and Tobacco. Alcohol Alert.

[b33-136-142] Perkins KA (1997). Combined effects of nicotine and alcohol on subjective, behavioral and physiological responses in humans. Addiction Biology.

[b34-136-142] Perkins KA, Fonte C, Grobe JE (2000). Sex differences in the acute effects of cigarette smoking on the reinforcing value of alcohol. Behavioural Pharmacology.

[b35-136-142] Pomerleau OF, Fertig JB, Allen JP (1995). Neurobiological interactions of alcohol and nicotine. Alcohol and Tobacco: From Basic Science to Clinical Practice.

[b36-136-142] Pomerleau OF (1998). Endogenous opioids and smoking: A review of progress and problems. Psychoneuroendocrinology.

[b37-136-142] Porjesz B, Begleiter H, Reich T (1998). Amplitude of visual P3 event-related potential as a phenotypic marker for a predisposition to alcoholism: Preliminary results from the COGA Project. Alcoholism: Clinical and Experimental Research.

[b38-136-142] Prescott CA, Kendler KS, Fertig JB, Allen JP (1995). Genetic and environmental influences on alcohol and tobacco dependence among women. Alcohol and Tobacco: From Basic Science to Clinical Practice.

[b39-136-142] Robinson TE, Berridge KC (1993). The neural basis of drug craving: An incentive-sensitization theory of addiction. Brain Research Reviews.

[b40-136-142] Rohsenow DJ, Monti PM, Colby SM (1997). Effects of alcohol cues on smoking urges and topography among alcoholic men. Alcoholism: Clinical and Experimental Research.

[b41-136-142] Saxon AJ, McGuffin R, Walker RD (1997). An open trial of transdermal nicotine replacement therapy for smoking cessation among alcohol- and drug-dependent inpatients. Journal of Substance Abuse Treatment.

[b42-136-142] Tiffany ST, Drobes DJ (1990). Imagery and smoking urges: The manipulation of affective content. Addictive Behaviors.

[b43-136-142] U.S. Department of Health and Human Services (DHHS) (1989). DHHS, Public Health Service, Centers for Disease Control, Center for Chronic Disease Prevention and Health Promotion, Office on Smoking and Health. Reducing the Health Consequences of Smoking: 25 Years of Progress. A Report of the Surgeon General.

[b44-136-142] Wise RA (1988). The neurobiology of craving: Implications for the understanding and treatment of addiction. Journal of Abnormal Psychology.

[b45-136-142] Zacny JP, Galanter M (1990). Behavioral aspects of alcohol-tobacco interactions. Recent Developments in Alcoholism: Vol. 8. Combined Alcohol and Other Drug Dependence.

